# Grafting Chitosan with Polyethylenimine in an Ionic Liquid for Efficient Gene Delivery

**DOI:** 10.1371/journal.pone.0121817

**Published:** 2015-04-13

**Authors:** Huiying Chen, Shaohui Cui, Yinan Zhao, Chuanmin Zhang, Shubiao Zhang, Xiaojun Peng

**Affiliations:** 1 State Key Laboratory of Fine Chemicals, Faculty of Chemical, Environmental and Biological Science and Technology, Dalian University of Technology, Dalian, Liaoning, China; 2 Key Laboratory of Biotechnology and Bioresources Utilization—The State Ethnic Affairs Commission-Ministry of Education, College of Life Science, Dalian Nationalities University, Dalian, Liaoning, China; RMIT University, AUSTRALIA

## Abstract

Modifying chitosan (CS) with polyethylenimine (PEI) grafts is an effective way to improve its gene transfection performance. However, it is still a challenge to conduct the grafting with fine control and high efficiency, particularly for the modification of water-insoluble CS. Herein, a novel method to graft CS with PEI (1.8 kDa, PEI-1.8) was developed by using ionic liquid 1-butyl-3-methyl imidazolium acetate ([BMIM]Ac) as a reaction solvent, water-insoluble CS as a reaction substrate and 1,1-carbonyldiimidazole (CDI) as a linking agent. The grafting reaction was greatly accelerated and the reaction time was largely shortened to 4 h by taking advantages of the good solubility of CS, the enhanced nucleophilicity of amino groups and the preferential stability of the activated complexes in the ionic liquid. The chitosan-*graft*-polyethylenimine (CS-*g*-PEI) products were characterized by ^1^H NMR, FTIR and GPC. PEI-1.8 was quantitatively grafted to CS through urea linkages, and the grafting degree (GD) was conveniently tuned by varying the molar ratios of PEI-1.8 to D-glucosamine units of CS in the range of 9.0 × 10^-3^ to 9.0 × 10^-2^. Compared with CS, the synthesized CS-*g*-PEI copolymers showed higher pDNA-binding affinity, which increased with the GD as shown in Agarose gel electrophoresis. The dynamic light scattering (DLS) experiment demonstrated that the CS-*g*-PEI/pDNA polyplexes had suitable particle sizes and proper ζ-potentials for cell transfection. The CS-*g*-PEI copolymer with a medium GD of 4.5% conferred the best gene transfection, with the efficiency 44 times of CS and 38 times of PEI-1.8 in HEp-2 cells. The cytotoxicity of CS-*g*-PEI was tested and found nearly as low as that of CS and much lower than that of PEI.

## Introduction

In recent decades, chitosan (CS), a natural linear cationic polysaccharide, has been widely used as drug carriers, wound dressings, anticoagulants and scaffolds for tissue engineering owing to its biocompatibility, biodegradability, mucoadhesion, low toxicity and immunogenicity [1–3]. Since the first study on using CS as a non-viral vector for pDNA delivery by Rolland *et al*. [4], increasing attention has been devoted to the application of CS in gene transfection [5–12]. The physicochemical and biological basis for the function of CS/nucleic acid has been partly elucidated. The proper pDNA-binding affinity and considerable buffering capacity are proposed to be necessary for CS to obtain an efficient transfection. The pDNA-binding affinity of CS can be modulated by adjusting the molecular weight (*M*_w_) and degree of deactylation (DDA) [13,14]. The considerable buffering capacity cannot be obtained by CS itself because of the poor proton absorption ability, which leads to the difficulty in the endosomal escape and the frustrated transfection [6,9]. Modifying CS with high proton buffering grafts to improve its proton buffering capacity has received much attention [15–27]. Polyethylenimine (PEI) is called “proton sponge”. It is one of the most efficient polymers for gene delivery due to the fast endosomal escape ability. Many researchers attempted to graft PEI to CS (chitosan-*graft*-polyethylenimine, CS-*g*-PEI) to enhance the transfection efficiency of CS [15–26]. For example, Wong *et al*. modified CS by polymerizing aziridine in the presence of water-soluble oligo-chitosan (*M*_n_ = 3400) at ambient temperature for 5 days. The as-prepared CS-*g*-PEI showed high gene transfection efficiency and low cytotoxicity in HepG2, HeLa, and primary hepatocyte cells [15]. Cho’s group prepared CS-*g*-PEI copolymer with periodate-oxidized CS (*M*_w_ = 100 kDa, DDA = 87.7%) and PEI (1.8 kDa, PEI-1.8). After 2 days reaction at 4°C, the obtained copolymer showed higher gene transfection efficiency and lower cytotoxicity than PEI (25 kDa, PEI-25) in Hela, 293T and HepG2 cell lines [16–18]. Li *et al*. synthesized CS-*g*-PEI copolymer in dithiodipropionic acid aqueous solution at 40°C for 24 h, via biocleavable disulfide linkages between chitosan (*M*_w_ = 10 kDa, 80 kDa, DDA = 85.3%) and PEI (0.8 kDa, PEI-0.8) [19]. The bio-reducible polymer/DNA polyplexes facilitated efficient release of DNA in the presence of 25 mM 1,4-dithiothreitol, mimicking the intracellular reductive environment [19]. All the work mentioned above used water-soluble CS oligosaccharides or water-soluble chitosan derivatives and low-*M*_w_-PEI for the grafting. However, it has been reported that CS with higher *M*_w_ of ca. 40 kDa will have the most suitable affinity to DNA for the efficient transfection [13]. In contrast, the CS with lower *M*_w_, such as water-soluble CS, and low-*M*_w_-PEI both have weaker binding affinity to DNA and consequently has insufficient transfection efficiency. Grafting low-*M*_w_-PEI to CS with suitable *M*_w_ and DDA should be an effective approach. Unfortunately, CS with *M*_w_ at ca. 40 kDa has very low solubility in neutral water and is difficult to be grafted in conventional ways [4,13]. In addition, as mentioned above, the reaction time for CS modification is very long, usually lasts for 24 h or several days. Such long time reaction readily leads to the gelation of reactant solution or the degradation of CS reactant, which results in the difficulty in controlling the grafting degree (GD), the structure of the graft copolymer, and the experiment reproducibility. Furthermore, in the aqueous reaction medium, water-intolerable reagent is hardly used. For example, Gao *et al*. synthesized N, N'-carbonyldiimidazole (CDI) linked CS-*g*-PEI for gene transfection in 20 ml 0.5% HAc with excess linking agent of CDI at room temperature over night [28]. The GD is difficult to be controlled because CDI is water intolerable and readily hydrolyzes before the grafting reaction.

Ionic liquid can be used as a good solvent for biological macromolecules, including CS, cellulose, wool keratin and silk fibroin [29–35]. Large *M*_w_ CS can be dissolved in ionic liquid 1-butyl-3-methylimidazolium acetate ([BMIM]Ac) at up to 50 wt% concentration by completely disrupting the hydrogen bonds in CS’s structure [35]. This makes it possible to modify CS in a homogeneous and efficient way in [BMIM]Ac. More importantly, it has been reported that ionic liquid could enhance the nucleophilicity of amines and increase the stability of the reagent activated complexes thus accelerating a nucleophilic substitution (S_N_) reaction [36,37]. This would shorten the reaction time and benefit the grafting control. Our study demonstrated that CS-*g*-PEI with a high GD could be developed in [BMIM]Cl and had good potential as an efficient and safe non-viral gene vector [31]. In the present report, we further disclosed an efficient and finely-controlled method to graft a given amount of low-*M*_w_-PEI to water-insoluble CS in [BMIM]Ac. The structures of the synthsized CS-*g*-PEI copolymers were identified by ^1^H NMR, FTIR and GPC characterizations. The buffering capacity, pDNA-binding affinity, polyplexes stability and gene transfection performance of the synthsized CS-*g*-PEI copolymers were evaluated. The results obtained in this work would provide valuable suggestions for the development of CS-based gene vectors.

## Materials and Methods

### Materials

Chitosan (*M*_n_ = 40 kDa, DDA = 85%) was supplied by Jinan Haidebei Marine Bioengineering Co. Ltd. (Jinan, China). PEI-1.8 was obtained from Aladdin Industrial Corporation (Shanghai, China). Branched PEI-25 was purchased from Sigma-Aldrich Chemical Co. (St. Louis, MO, USA). CDI was supplied by Beijing Chemicals Co. Ltd. (Beijing, China). Ionic liquid [BMIM]Ac was prepared from [BMIM]Br and AgAc according to the method developed by Zhao *et al*. [38]. Cells were purchased from American Type Culture Collection (ATCC Shanghai Representative Office, Shanhai, China). Luciferase activity assay kits and the protein assay kits were obtained from Biyuntian Biologic Co. Ltd. (Nanjing, China). Dulbecco’s modified eagle’s medium (DMEM) and trypsin were obtained from Gibco BRL (Gaithersberg, MD, USA). Fetal bovine serum (FBS) was purchased from Sijiqing Biologic Co. Ltd. (Hangzhou, China). The plasmid DNA (pDNA) was amplified in Escherichia coli cells and purified using the endo-free Qiagen kit (Qiagen, Valencia, CA, USA) to remove the bacterial endotoxins. All the organic solvents used were of analytical grade and dehydrated before use.

### Synthesis and characterization of CS-*g*-PEI

**Fig 1:**
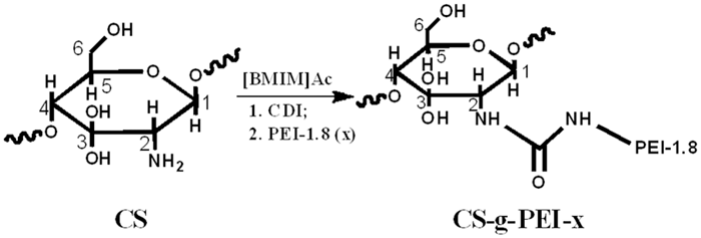
Grafting PEI-1.8 to CS via CDI to synthesize CS-*g*-PEI in [BMIM]Ac.

CS-*g*-PEI copolymer was synthesized following the route as shown in Fig 1. First, 0.2 g CS powder was added to a flask containing 20 ml ionic liquid [BMIM]Ac and then heated to 80°C in a N_2_ atmosphere. When CS was completely dissolved in [BMIM]Ac as confirmed by a polarizing microscopy, CDI with equimolar amount of CS’s amino groups was added. After reaction at 80°C for 2 h, 9.0 × 10^-3^, 1.8 × 10^-2^, 4.5 × 10^-2^ and 9.0 × 10^-2^ equiv of PEI-1.8 per CS D-glucosamine unit were added respectively, under stirring and reacted at 80°C for another 2 h in the N_2_ atmosphere to get CS-*g*-PEI with different GD. After cooling to room temperature, water was added to the reaction mixture to precipitate the products. Then, the products obtained by filtration were transferred into a dialysis tube (molecular weight cut-off, MWCO~3500) and purified in deionized water for 3 days. Finally, the products were lyophilized to give out light golden brown powders, which were soluble in phosphate buffered saline (PBS). The [BMIM]Ac solvent was recovered by evacuating the water from the filtrate. The products were denominated as CS-*g*-PEI-0.9, CS-*g*-PEI-1.8, CS-*g*-PEI-4.5 and CS-*g*-PEI-9.0 respectively, based on the molar ratio of PEI-1.8 to CS’s D-glucosamine unit added in the reactants (9.0 × 10^-3^, 1.8 × 10^-2^, 4.5 × 10^-2^ and 9.0 × 10^-2^).

The structures of the prepared CS-*g*-PEI copolymers were characterized by ^1^H NMR and FTIR. ^1^H NMR measurements were carried out at 25°C and the solvents of D_2_O doped with CD_3_COOD were used (Mercury plus400, Agilent-Varian, USA). FTIR spectra were recorded on KBr pellets in a range from 4000 to 400 cm^-1^ at atmosphere (IR Prestige-21, Shimadzu, Japan). The *M*_w_ of CS-*g*-PEI copolymers were measured by GPC (Malvern, Viscotek TDAmax, UK), using 0.5 M ammonium acetate (pH = 5.5) as the mobile phase.

The buffering capacity of PEI-25, PEI-1.8, CS and CS-*g*-PEI copolymers were determined by acid-base titration assay over the pH values ranging from 10 to 3 as described previously by Benns *et al*. [39,40]. Briefly, the polymer was dissolved in 150 mM NaCl solution. The pH of the solution was tuned to 10 with 0.1 M NaOH and then 0.1 M HCl solution was added in increments of 200 μl. The pH values were recorded on a pH meter. The buffering capacity was calculated according to the volume of HCl solution needed to decrease the pH value of polymer solution from 7.5 to 4.0.

### Preparation and characterization of CS-*g*-PEI/pDNA polyplexes

All CS-*g*-PEI/pDNA polyplexes were freshly prepared by a coacervation method which was widely used [41,42]. Briefly, a solution of polymer was added to a solution of pDNA at specific N/P ratio under gentle vortex, and incubated for 30 min at room temperature before use. Unless specified otherwise, the N/P ratio of polymer to DNA in this paper is weight ratio.

To investigate the binding affinity of CS-*g*-PEI copolymers to pDNA, the CS-*g*-PEI/pDNA polyplexes with different N/P ratios were loaded on a 1% agarose gel with Tris-acetate (TAE) running buffer and subjected to electrophoresis at 90 V and 50 mA for 60 min (0.1 mg of pDNA per hole, the N/P ratio varying from 1 to 6). The fluorescence of the intercalated dye (ethidium bromide) was measured using a gel imaging system (Syngene, UK). To evaluate the stability of the polyplexes in serum, the freshly prepared polyplex (at the N/P ratio of 6) solutions and FBS solutions were mixed in Eppendorf tubes. After incubating at 37°C for 30 min, the stability of the polyplexes in serum was evaluated by electrophoresis.

Hydration particle size and ζ-potential of the polyplexes were measured in triplicate on a dynamic light scattering (DLS, Nano ZS 90, Malvern, UK) with 90° scattering angles at 25°C. The CS-*g*-PEI/pDNA polyplexes were prepared in water at the N/P ratio of 6.

### Cell transfection

To investigate the gene delivery efficiency of the synthesized copolymers, cell transfections were performed in human epithelial type 2 cells (HEp-2, purchased from American Type Culture Collection, ATCC) *in vitro* with pGFP-N2 and pGL3 as report genes. One day before transfection, 0.5 × 10^4^ ~ 2 × 10^4^ cells were seeded per well in 500 μl growth medium (DMEM) until the required cell number was obtained 80% confluence at the time of transfection. All polyplexes were freshly prepared by the method mentioned above. The N/P ratios of the polyplexes were fixed at 6 for pGFP-N2 report gene, and varied from 1 to 10 for pGL3 report gene. The polyplexes were added to the 24-well plate and incubated for 5 h in DMEM without serum and antibiotics at 37°C under 5% CO_2_ atmosphere. Then DMEM was replaced with the fresh DMEM with 10% serum. After 48 h, the transfection efficiency was analyzed by the green fluorescence images through an inverted fluorescence microscope when pGFP-N2 was used as report gene. Luciferase activity for pGL3 gene was measured after the lysis buffer was added into each well of 96-well plate and incubated for 5 min at room temperature after 48 h post-transfection. The protein concentrations of cell lysates per well were determined using BCA protein assay reagent, and then the transfection efficiency was obtained as the relative luciferase activity. Data were expressed as relative light units (RLU) per mg protein. PEI-1.8 and PEI-25 were used as controls.

### Cytotoxicity assays

Cytotoxicity was evaluated using the 3-(4,5-dimethylthiazol-2-yl)-2,5-diphenyltetrazolium bromide (MTT) assay method. Before cell viability assay, HEp-2 cells were seeded in a 96-well plate at a density of 5 × 10^3^ cells/well. After incubation for 12 h, polyplexes prepared as mentioned above were added with 100 μl serum-free DMEM medium to replace the culture medium. After incubation at 37°C under a 5% CO_2_ atmosphere for 24 h, 20 μl of MTT solution (5 mg/ml) was added to each well and incubated for another 4.5 h. The cultured media was then replaced by 150 μl DMSO. The wells treated with DMEM medium only were used as control. The optical density (OD) was measured on an enzyme microplate reader (Tecan Sunrise) at 570 nm. The cell viability was calculated as follows: cell viability = OD570 (sample)/OD570 (control) × 100%. All the experiments were carried out in triplicate to ensure the reproducibility.

## Results and Discussion

### Synthesis and characterization of the graft copolymers

**Fig 2:**
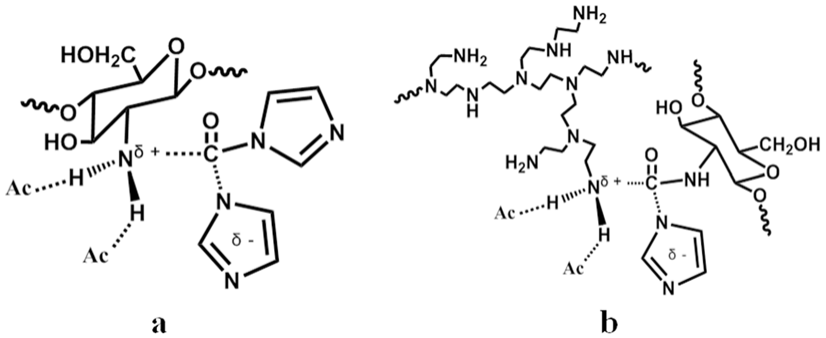
Ionic liquid anions hydrogen-bonding with ammonium ions in the activated complex. (a) activated complex of CS and CDI, (b) activated complex of PEI and CS-CDI intermediate.

CS-*g*-PEI copolymers were synthesized in ionic liquid [BMIM]Ac using CDI as a linking agent. As shown in Fig 1, CS-*g*-PEI was synthesized through urea linkages. First, CS reacted with CDI at amino groups forming an active intermediate through S_N_ reaction; PEI-1.8 then reacted with the intermediate of CS and CDI to obtain the CS-*g*-PEI copolymers also through S_N_ reaction. In the ionic liquid, the S_N_ reactions of CS and CDI, CS-CDI intermediate and PEI were significantly accelerated and completed in 2 h, respectively. Compared to the reaction conducted in the literature [15–19], the reaction rate in the present study was accelerated by 6–30 times, with the reaction time shortened from 5 days or 24 h to 4 h. On one hand, this high efficiency should be attributed to the homogeneous reaction condition after CS dissolving in [BMIM]Ac. On the other hand, the nucleophilicity of amino groups of CS and PEI would be enhanced in [BMIM]Ac, and this would further accelerate the reaction [36]. Furthermore, the activated complexes (as shown in Fig 2) of the S_N_ reactions would be preferentially stabilized by the hydrogen-bond-accepting anion of the ionic liquid [36]. This effect would result in a rather dramatic increase in the rate of the reaction by decreasing the reaction activation energy. Therefore, the S_N_ grafting reaction was completed in a short time, which benefited the GD control.

**Fig 3:**
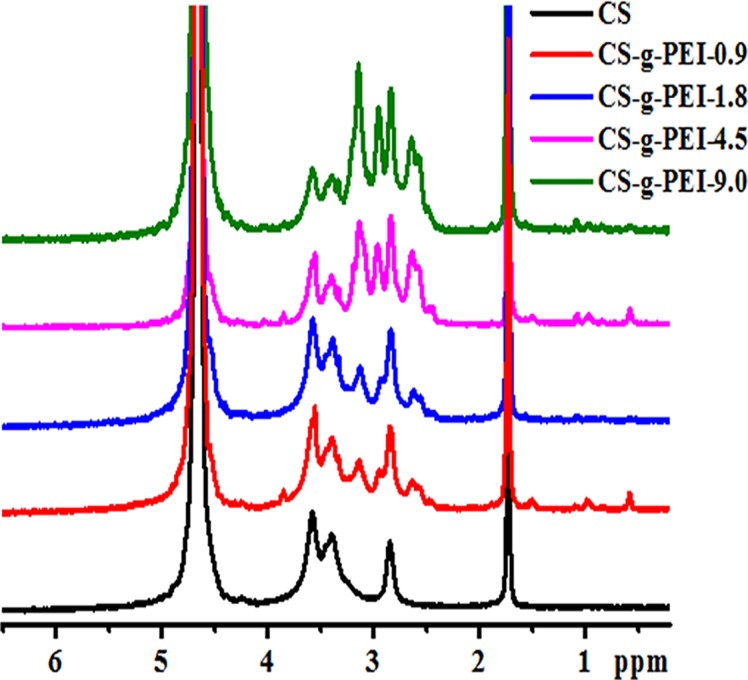
^1^H NMR spectra of CS and CS-*g*-PEI copolymers with different grafting degrees.

The CS-*g*-PEI copolymers with different GD were obtained by changing the molar ratios of PEI-1.8 to CS’s D-glucosamine unit from 9.0 × 10^-3^ to 9.0 × 10^-2^. Fig 3 shows the ^1^H NMR spectra of the synthesized copolymers. The proton peaks of CS’s D-glucosamine unit (H-3, H-4, H-5, H-6, ref Fig 1) appeared at δ = 3.1–3.8 ppm. The peaks at δ = 2.5–3.2 ppm belonged to PEI-1.8 (–NHCH_2_CH_2_–), indicating that PEI-1.8 was successfully grafted to the CS’s chain [15,17]. The relative intensity at δ = 2.5–3.2 ppm increased with the amount of PEI-1.8 grafted to CS. By comparing the intensity of the peaks at δ = 2.5–3.2 ppm and δ = 3.1–3.8 ppm, the degrees of PEI-1.8 per CS D-glucosamine unit could be calculated [19]. They were 0.9%, 1.6%, 4.5% and 8.6% for CS-*g*-PEI-0.9, CS-*g*-PEI-1.8, CS-*g*-PEI-4.5 and CS-*g*-PEI-9.0, respectively. The GD linearly increased with the amount of PEI-1.8 fed in the reactants. The GD almost equaled to the molar ratio of PEI-1.8 fed, indicating that PEI-1.8 was quantitatively grafted to CS in the range of present investigation. By changing the addition amount of PEI-1.8, grafting-degree-controllable synthesis of CS-*g*-PEI copolymers could be performed in [BMIM]Ac ionic liquid.

**Fig 4:**
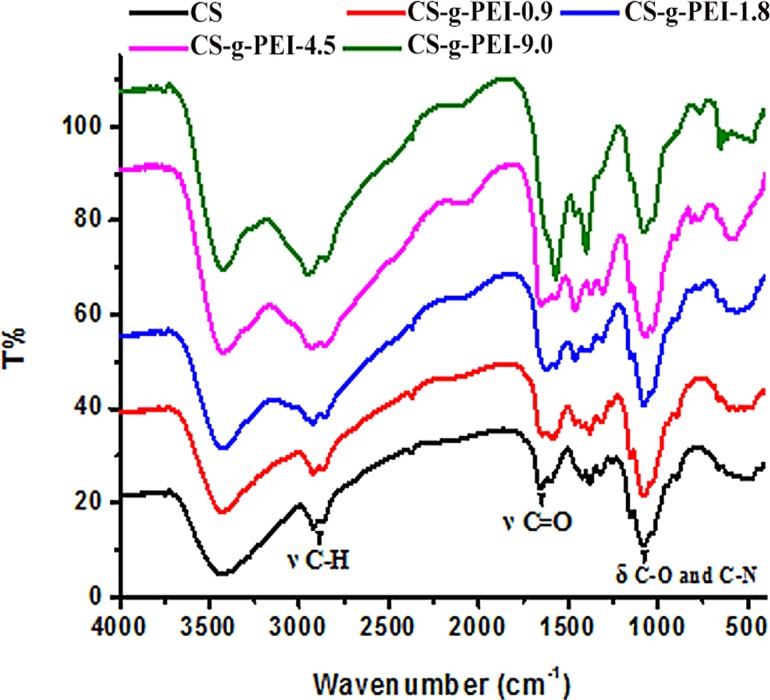
FTIR spectra of CS and CS-*g*-PEI copolymers with different grafting degrees.

The FTIR spectra of CS and CS-*g*-PEI copolymers are shown in Fig 4. The absorption of ν(O-H) and ν(N-H) at 3450–3200 cm^−1^, ν(C-H) at 2928 and 2886 cm^-1^, ν(C = O NH_2_) at 1656 and 1597 cm^−1^, and ν(C-O-C) at 1155 cm^-1^ in the FTIR spectra were attributed to CS D-glucosamine units. After grafting PEI-1.8, a new peak appeared at 1590 cm^-1^, which should be attributed to the carboxyl in urea group. In addition, the intensity of peaks at 2928, 2886 and 1412 cm^-1^ were strengthened due to the presence of-CH_2_CH_2_NH- moiety of PEI-1.8 grafts [19,43]. Moreover, the intensity of these peaks increased with the GD. These FTIR spectra further confirmed that CS-*g*-PEI copolymers with different GD were obtained in ionic liquid [BMIM]Ac.

The *M*_w_ of CS-*g*-PEI copolymers measured by GPC were listed in Table 1. The GD based on *M*_w_ was 0.8%, 1.7%, 4.3% and 8.1% for CS-*g*-PEI-0.9, CS-*g*-PEI-1.8, CS-*g*-PEI-4.5 and CS-*g*-PEI-9.0, respectively. These values were well-matched with the GD derived from ^1^H NMR.

**Table 1:** Molecular weight (*M*_w_) of CS-*g*-PEI copolymers measured by GPC.

Poymers	Molecular Weight (*M*_w_, kDa)	Grafting degree (%)
**CS**	64	--
**CS-*g*-PEI-0.9**	70	0.8
**CS-*g*-PEI-1.8**	76	1.7
**CS-*g*-PEI-4.5**	95	4.3
**CS-*g*-PEI-1**	122	8.1

**Fig 5:**
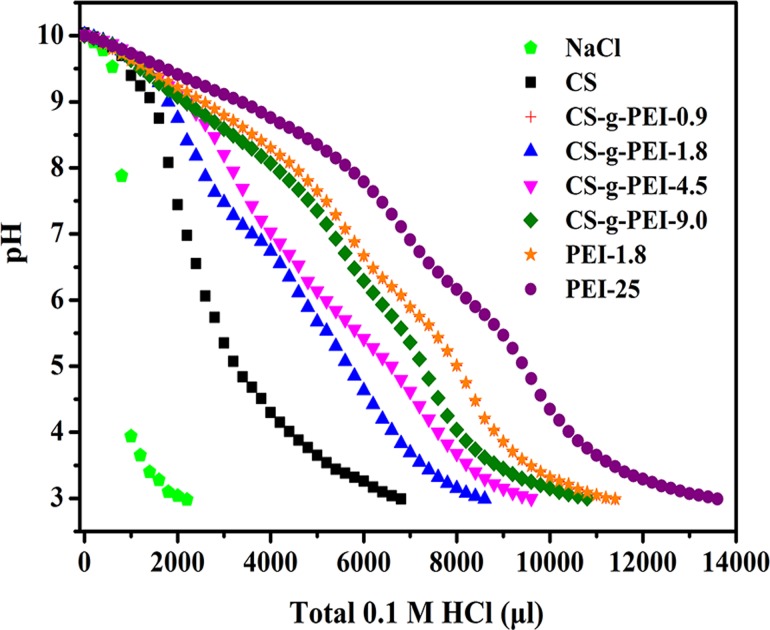
Buffering capacity in 150 mM NaCl solutions. NaCl blank (⬟), CS (■), CS-*g*-PEI-0.9 (+), CS-*g*-PEI-1.8 (▲), CS-*g*-PEI-4.5 (▼), CS-*g*-PEI-9.0 (◆), PEI-1.8 (★) and PEI-25 (●).

Many studies have demonstrated that the buffering capacity of polymeric gene delivery vectors plays an important role in efficient endosomal escape and transfection [43–46]. Conjugating CS with PEI has been proven an effective approach to improve the buffering capacity of CS-based vectors [19]. To determine the buffering capacity of synthesized CS-*g*-PEI copolymers, an acid-base titration assay was performed in 150 mM NaCl solution by 0.1 M HCl in increments of 200 μl to decrease the pH from 10 to 3. The results are shown in Fig 5. The buffering capacity of CS was much lower than that of PEI and significantly improved by PEI grafts. The buffering capacity was quantified according to the volume of HCl solution needed to decrease the pH value of polymer solution from 7.5 to 4, as shown in the following equation:

$$Buffering\;capacity=\left(\Delta V\times 0.1M\times 36.5g\cdot mol^{-1}\times 10^{-3}\right)/W\times 100\%,$$

where ΔV is the volume of HCl solution (μl), and W is the total weight of polymer (mg). The calculated buffering capacities of NaCl, CS, PEI and CS-*g*-PEI copolymers were listed in Table 2. The buffering capacity of CS was improved by PEI grafts and increased with the GD. When the GD increased to 4.5%, the buffering capacity reached the maximum of 24.3%, comparable to that of PEI-25.

**Table 2:** Buffering capacity of CS, CS-g-PEI copolymers, PEI-1.8 and PEI-25 measured by acid-base titration.

Poymers	ΔV (μl)	W (mg)	Buffering capacity (%)
**NaCl**	200	60	1.3
**CS**	2400	60	14.6
**CS-*g*-PEI-0.9**	3200	60	19.5
**CS-*g*-PEI-1.8**	3600	60	21.9
**CS-*g*-PEI-4.5**	4000	60	24.3
**CS-*g*-PEI-1**	3600	60	21.9
**PEI-1.8**	3600	60	21.9
**PEI-25**	4000	60	24.3

All the results suggested that PEI-1.8 could be grafted to CS at a controlled GD in a short reaction time in [BMIM]Ac. The calculated GD values of the synthesized CS-g-PEI copolymers based on ^1^H NMR and GPC analysis agreed well with each other, suggesting that the amount of PEI-1.8 grafted to CS was quite close to the addition amount of PEI-1.8. That is to say, using [BMIM]Ac as the non-aqueous solvent and CDI as the linking agent, PEI-1.8 can be grafted to CS at a finely controlled GD in the homogeneous solution.

### Polyplexes of CS-*g*-PEI copolymers and pDNA

**Fig 6:**
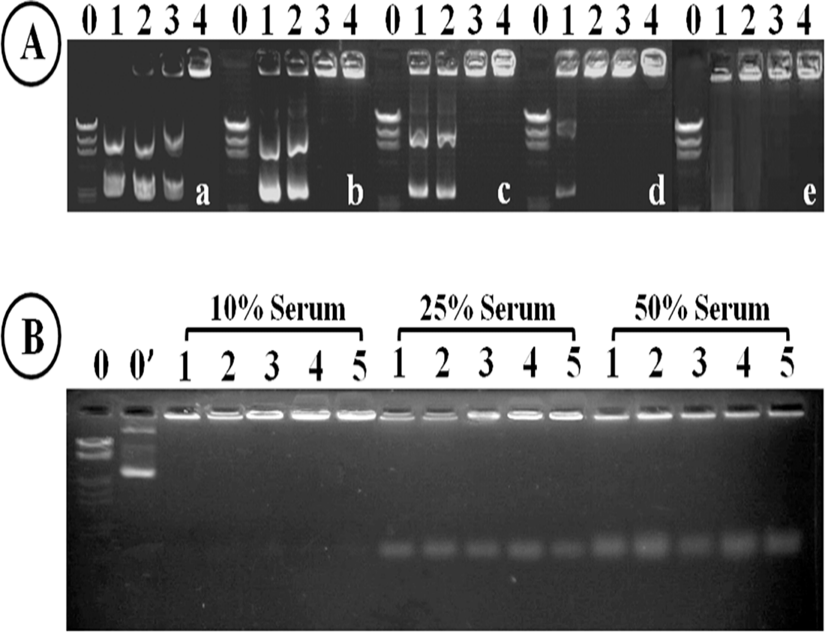
Agarose gel electropherogram. (A) Polyplexes at various N/P ratios without serum: (a) CS/pDNA, (b) CS-*g*-PEI-0.9/pDNA, (c) CS-*g*-PEI-1.8/pDNA, (d) CS-*g*-PEI-4.5/pDNA, and (e) CS-*g*-PEI-9.0/pDNA. Lane 0: DNA markers, lanes1-4: N/P ratios of 1, 2, 4 and 6, respectively. (B) Polyplexes at the N/P ratio of 6 in 10%, 25%, 50% serum, respectively. Lane 0: DNA markers, lane 0’: pDNA, lane 1: CS/pDNA, lane 2: CS-*g*-PEI-0.9/pDNA, lane 3: CS-*g*-PEI-1.8/pDNA, lane 4: CS-*g*-PEI-4.5/pDNA, lane 5: CS-*g*-PEI-9.0/pDNA.

The binding affinity of CS-*g*-PEI copolymers with pDNA was evaluated on gel electrophoresis. Fig 6A shows that the migration of pDNA was completely retarded at lower N/P ratios by CS-*g*-PEI copolymers compared with CS. CS completely retarded pDNA at the N/P ratio of 6. When PEI was grafted to CS, pDNA could be completely retarded at lower N/P ratios of 4, 4, 2 and 1 by CS-*g*-PEI-0.9, CS-*g*-PEI-1.8, CS-*g*-PEI-4.5 and CS-*g*-PEI-9.0, respectively. With the increase of the GD, the retardation ability of CS-*g*-PEI copolymers increased in an order of CS<CS-*g*-PEI-0.9<CS-*g*-PEI-1.8<CS-*g*-PEI-4.5<CS-*g*-PEI-9.0. It was reported that the pDNA-binding affinity of low-*M*_w_-PEI was insufficient [47]. Accordingly, the improved pDNA-binding affinity should be mostly contributed to the extended conformation of the synthesized CS-*g*-PEI copolymers [47].

Polyplexes would be dissociated by the molecules with negative charges in serum [48]. To evaluate the stability of the polyplexes in serum, FBS was mixed with freshly prepared polyplexes at the N/P ratio of 6. After incubating at 37°C for 30 min, the mixtures were loaded on a 1% agarose gel and subjected to electrophoresis. As shown in Fig 6B, there was no pDNA released from the polyplexes in 10% serum. This demonstrated that the polyplexes were stable in 10% serum. When the concentration of serum increased to 25%, a little amount of pDNA was dissociated from the polyplexes. When the concentration of the serum reached 50%, more pDNA dissociated from the polyplexes. Therefore, it could be concluded that the CS-*g*-PEI/pDNA polyplexes were stable when the serum concentration was less than 10%.

**Fig 7:**
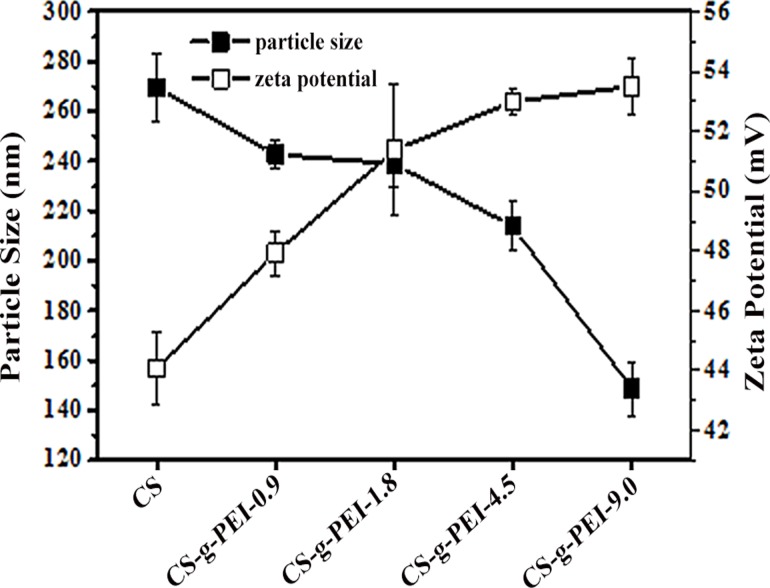
Particle sizes and ζ-potentials of CS/pDNA and CS-*g*-PEI/pDNA polyplexes (N/P = 6) determined by DLS.

Particle size and ζ-potential of a polyplex are very important for the polyplex’s uptake by cells [49]. Particle size influences the internalization of a polyplex into cells, while ζ-potential has close relationship with the stability of a polyplex in the circulation system. Fig 7 shows the particle sizes and ζ-potentials of CS and CS-*g*-PEI polyplexes at the N/P ratio of 6. The particle sizes of all the polyplexes were in a range of 270–150 nm, and decreased with the increase of the GD of CS-g-PEI copolymers. When PEI-1.8 was grafted to CS, more amino groups of CS-*g*-PEI copolymer could interact with negatively charged pDNA. The enhanced intermolecular binding interaction would result in a decrease in the particle size of the polyplex [47]. ζ-potential reflects the net charge of a polyplex. As shown in Fig 7, CS and CS-*g*-PEI polyplexes were all positively charged at the N/P ratio of 6. The ζ-potentials of the polyplexes were higher than 40 mV, indicating that there was net electrostatic repulsive force to prevent polyplexes from aggregation, which accounted for the stability of the polyplexes [50]. Furthermore, the ζ-potentials of CS-*g*-PEI/pDNA polyplexes increased with the GD of CS-*g*-PEI copolymers. This indicated that PEI grafts in the CS-*g*-PEI copolymers could further neutralize the negative charges of pDNA and increase the ζ-potentials of the polyplexes. The positive surface charge of the polyplexes was also helpful for binding anionic cell, which consequently facilitated the polyplexes to enter into cells via the endocytosis [40,46].

Evidently, grafting low-*M*_w_-PEI to CS enhanced its pDNA-binding affinity. The pDNA retardation ability of CS-*g*-PEI copolymers increased with the GD and the CS-*g*-PEI/pDNA polyplexes were stable in 10% serum. In addition, with the increase of the GD, CS-*g*-PEI/pDNA polyplexes had more positive charge and smaller size suitable for the cell transfection.

### Cell transfection

**Fig 8:**
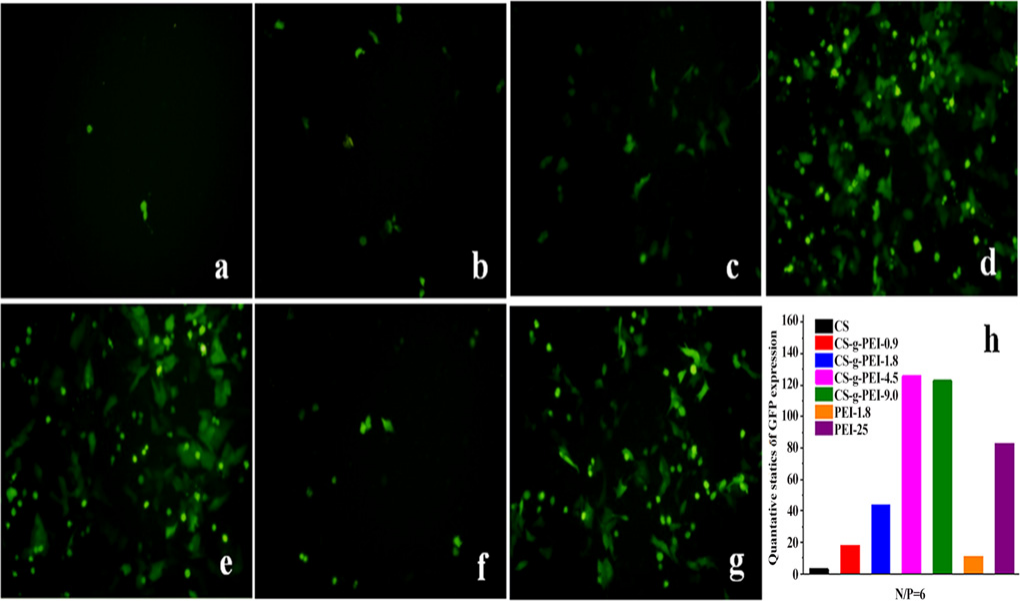
Fluorescence images of HEp-2 cells exposed to the polyplexes (N/P = 6) after transfection of 24 h. (a) CS/pDNA, (b) CS-*g*-PEI-0.9/pDNA, (c) CS-*g*-PEI-1.8/pDNA, (d) CS-*g*-PEI-4.5/pDNA, (e) CS-*g*-PEI-9.0/pDNA, (f) PEI-1.8/pDNA and (g) PEI-25, using pGFP-N2 as report gene. Relative quantitative GFP expression efficiency of polyplexes was shown (h).

To evaluate the gene delivery efficiency of the synthesized CS-*g*-PEI copolymers against HEp-2 cells, pGFP-N2 was used as report gene and the images of transfected fluorescent cells were taken with a fluorescence inverted microscope. Fig 8 shows the green fluorescent images of cells transfected by CS/pDNA and CS-*g*-PEI/pDNA polyplexes at the N/P ratio of 6 for 24 h, with PEI-1.8 and PEI-25 as controls. The density and intensity of fluorescent cells increased with the GD of CS-*g*-PEI copolymers. In contrast, little green fluorescence was observed by CS at 24 h post-transfection. The quantitative statistic of GFP expression efficiency of polyplexes is shown in Fig 8(h). The results demonstrated that the gene delivery ability of CS was significantly improved by grafting PEI-1.8 to CS, which was consistent to that reported by Wong *et al*. [15], Jiang *et al*. [17], Pezzoli *et al*. [22], and Liu *et al*. [48]. The high amine content in CS-*g*-PEI copolymers would contribute to the easier escape of the polyplexes due to the high buffering capacity, and thus an improved transfection [44].

**Fig 9:**
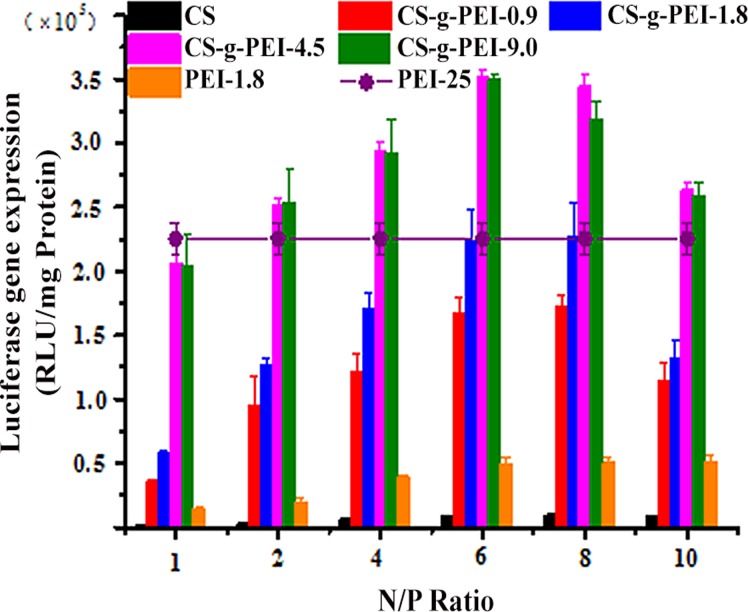
Luciferase expression in HEp-2 cells. Transfection with CS/pDNA and CS-*g*-PEI/pDNA polyplexes at various N/P ratios using pGL3 as report gene, PEI-25 as positive control.

Luciferase activity assays were also conducted to quantitatively investigate the transfection efficiency of CS-*g*-PEI copolymers, using pGL3 as report gene. pGL3 was delivered by the copolymers into HEp-2 cells at various N/P ratios ranging from 1 to 10. PEI-25/pDNA polyplex at the optimal N/P ratio of 1.3 (molar ratio of PEI’s amine groups to pDNA’s phosphates equaled to 10) was used as control [15,43]. The transfection efficiency was analyzed by the luciferase activity which was expressed as relative light units per mg protein (RLU/mg) at 48 h post-transfection. As shown in Fig 9, transfection efficiency of CS-*g*-PEI copolymers increased remarkably with the GD. The transfection efficiency of CS-*g*-PEI-4.5 and CS-*g*-PEI-9.0 was similar and reached the summit at the N/P ratio of 6. The transfection efficiency of CS-*g*-PEI-4.5 and CS-*g*-PEI-9.0 was nearly 44 times of CS and 38 times of PEI-1.8 at the N/P ratio of 6. When compared with PEI-25, the transfection efficiency of CS-*g*-PEI-4.5 and CS-*g*-PEI-9.0 increased by 3.6 times at the N/P ratio of 6. When the N/P ratios were higher than 8, the transfection efficiency decreased. This was most probably because the compact structure of the polyplexes did not facilitate the release of pDNA in the cells. The transfection efficiency of CS-*g*-PEI-0.9 and CS-*g*-PEI-1.8 was not as high as that of CS-*g*-PEI-4.5 and CS-*g*-PEI-9.0, probably due to the low GD. In contrast, the transfection efficiency of PEI-1.8 was much low in the tested concentration. It could be concluded that the transfection efficiency of CS was improved by PEI-1.8 grafting. The results were consistent with that of pGFP-N2 assays, further demonstrating that the gene delivery ability of CS was significantly improved by grafting PEI-1.8 to CS and the grafting synthesis in ionic liquid [BMIM]Ac was efficient.

### Cytotoxicity assays

**Fig 10:**
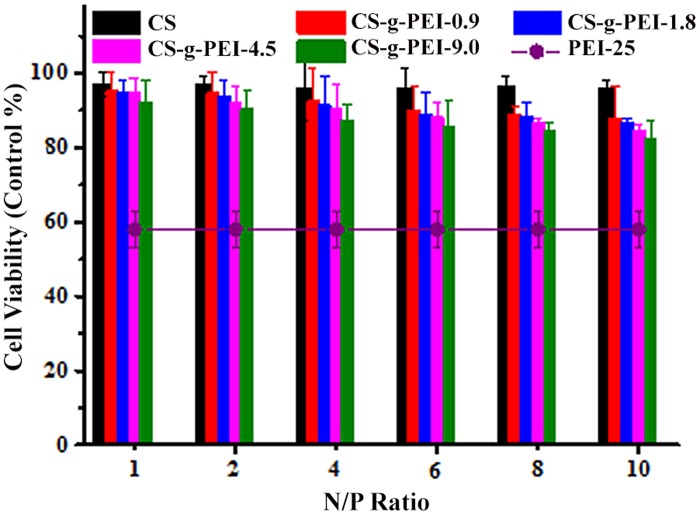
Viability of HEp-2 cells after incubation with CS/pDNA and CS-*g*-PEI/pDNA polyplexes for 24 h at various N/P ratios. Cells with no treatment used as control; PEI-25 as negative control.

Safety is a principal criterion on gene delivery system for clinical application [17]. We evaluated the cytotoxicity of the as-synthesized CS-*g*-PEI copolymers with MTT assays against HEp-2 cells, with PEI-25/pDNA polyplex at the N/P ratio of 1.3 (molar ratio of 10) as control. Fig 10 shows that CS-*g*-PEI copolymers had much lower cytotoxicity as compared with PEI-25. Also, it was less cytotoxic than PEI-1.8. Over 80% cell viability was observed in the cases of CS and CS-*g*-PEI copolymers at the tested N/P ratios. However, only less than 60% cells survived with PEI-25. The cytotoxicity of cationic polymers was probably caused by polymer aggregation on cell surfaces to impair membrane functions [15,17]. Also, the primary amine interference disrupted intracellular protein kinase activity [45]. It was suggested that CS-*g*-PEI copolymers would be degraded into chitosan units and low molecular weight PEI which was less toxic in cells [15]. This would be the reason why CS-*g*-PEI copolymers had low cytotoxicity and good biocompatibility as compared with PEI-25. In addition, the cytotoxicity of CS-*g*-PEI copolymers in Fig 10 increased slowly with the increase of the GD, but all were in the tolerable range.

The MTT assays demonstrated that CS-*g*-PEI copolymers with different GD were much safer than PEI-25. Taking the transfection efficiency into consideration, CS-*g*-PEI-4.5 with a medium GD of 4.5% was the best among the as-synthesized CS-*g*-PEI copolymers.

## Conclusions

The synthesis of CS-*g*-PEI copolymers with controllable GD was developed in ionic liquid [BMIM]Ac. The ionic liquid significantly promoted the S_N_ reactions between CS and CDI, PEI and CS-CDI intermediate, with the reaction time shortened 6–30 times as compared with that in the aqueous solution. The GD could be conveniently tuned by varying the amount of PEI-1.8 charged thanks to the nearly quantitative reaction of PEI-1.8 and CS in [BMIM]Ac, as the molar ratio of PEI-1.8 to D-glucosamine unit of CS ranging from 9.0×10^-3^ to 9.0×10^-2^. The ^1^H NMR, FTIR, and GPC analyses confirmed that PEI was grafted to CS via the urea linkage. The obtained CS-*g*-PEI copolymers showed improved buffering capacity and pDNA-binding affinity, which increased with the GD of the copolymers. DLS showed that the particle sizes of all the polyplexes at the N/P ratio of 6 were in a range of 270–150 nm and decreased with the increase of the GD; the ζ-potentials of the poplyplexes were positive and increased with the increase of the GD. CS-*g*-PEI copolymer at a medium GD of 4.5% conferred the most excellent gene transfection against HEp-2 cells with low cytotoxity. Modifying CS in an ionic liquid was a green and efficient method particularly for the water-insoluble and water-intolerable reactants. This endorsed CS-based non-viral gene delivery vectors an encouraging prospective for gene delivery.
